# Self-Reported ADHD Among Portuguese Medical Residents: Medication Use, Psychiatric Comorbidities, and Perceived Professional Impact

**DOI:** 10.7759/cureus.117586

**Published:** 2026-10-07

**Authors:** João P Azenha, Margarida Cândido, Joana T Domingos, Raquel L Medinas, Daniel E Sousa

**Affiliations:** 1 Psychiatry and Mental Health, Unidade Local de Saúde de Lisboa Ocidental, Lisbon, PRT

**Keywords:** adhd screening, adult adhd, medical residents, self-diagnosis, stimulant medication

## Abstract

Background

ADHD is increasingly recognised in medical trainees, yet data from Portuguese residency programmes are lacking.

Objectives

This study aimed to assess the proportion of self-reported diagnosed or suspected ADHD among Portuguese medical residents, characterise medication use patterns, identify psychiatric comorbidities, and evaluate perceived professional impact.

Methods

Cross-sectional, observational study based on an anonymous online questionnaire distributed to medical residents across multiple Portuguese training centres between March and June 2026 (n = 225). ADHD symptoms were assessed using the Portuguese version of the Adult ADHD Self-Report Scale version 1.1 (ASRS-v1.1).

Results

A total of 225 residents completed the questionnaire. Diagnosed or suspected ADHD was self-reported by 37.3% (n = 84) of participants. Among this group, 66.7% (n = 56) reported that ADHD was first identified after commencing residency, and 56.0% (n = 47) reported no formal evaluation. Medication use was reported by 25.8% (n = 58). Among medication users, 32.8% (19/58) reported obtaining medication from a colleague or friend, through self-prescription, or without a formal prescription. Psychiatric comorbidities were significantly more prevalent in the self-reported ADHD group than in participants not reporting ADHD (61/84, 72.6% vs 69/141, 48.9%; p < 0.001). Professional impact was perceived as negative by 83.3% (n = 70) of this group.

Conclusions

Portuguese medical residents reported high rates of diagnosed or suspected ADHD, frequent medication use outside conventional prescribing pathways, and substantial perceived professional impairment. These findings highlight the need for structured diagnostic pathways and institutional support within residency training programmes.

## Introduction

ADHD is a neurodevelopmental condition characterised by developmentally inappropriate and impairing patterns of inattention, hyperactivity and/or impulsivity [1]. ADHD can persist into adulthood and is associated with clinically significant impairment across academic, occupational and interpersonal domains [1]. International epidemiological studies estimate the prevalence of adult ADHD at approximately 2.5%-3.1%, although estimates vary according to diagnostic criteria, assessment method and population characteristics [1,2]. Longitudinal evidence indicates that clinically significant symptoms persist into adulthood in a majority of affected individuals, often with a phenotypic shift towards predominantly inattentive and dysexecutive features as overt hyperactivity attenuates with age [3]. Adult ADHD remains under-recognised, particularly in high-functioning individuals whose symptoms may be partially compensated by intellectual ability, structured environments or adaptive coping strategies until occupational demands exceed these compensatory mechanisms [4].

The relationship between ADHD symptom expression and environmental demands is well established. Executive dysfunction, encompassing impairments in working memory, attentional control, planning, and emotional regulation, tends to become most apparent in high-load contexts requiring sustained self-direction and adaptive flexibility [5]. Medical residency training may represent such a high-demand environment, given its complex clinical responsibilities, administrative burden, irregular schedules, and examination demands, which may make pre-existing attentional and executive difficulties more apparent [4].

The available literature on ADHD in medical trainees remains limited, particularly among residents. Studies in medical students report highly variable prevalence estimates, ranging from approximately 1.7% based on self-report to 38.9% using Adult ADHD Self-Report Scale (ASRS)-based screening, with substantial variation according to assessment methodology [6]. Recent work has suggested that ADHD in medical learners and physicians may be underdiagnosed and undertreated, partly because of stigma, concerns regarding professional scrutiny, and the misconception that successful progression through medical training is incompatible with clinically significant ADHD [4]. Among residents and fellows referred to academic or professional difficulty, ADHD has been associated with impaired organisational efficiency, reduced engagement in training, repeated errors and difficulties with examination or training progression [7].

A further concern in medical training environments is the potential overlap between ADHD symptoms, psychiatric comorbidity and stimulant use. Adults with ADHD frequently present with comorbid anxiety, depressive symptoms and sleep disturbances, all of which may both mimic and exacerbate attentional difficulties [8,9]. This overlap complicates diagnostic assessment and increases the risk of self-misattribution when formal evaluation is not performed [9]. At the same time, prescription stimulants such as methylphenidate and amphetamine derivatives are established treatments for ADHD when appropriately prescribed and monitored, but their non-medical or unsupervised use for cognitive enhancement has been described among university and medical student populations [10].

The present study aimed to characterise the proportion of self-reported diagnosed or suspected ADHD and the rate of positive screening on the Portuguese version of the ASRS (v1.1) among medical residents undertaking specialty training in Portugal. Secondary objectives included describing patterns of formal clinical evaluation, medication use and prescribing pathways, psychiatric comorbidities, and the perceived functional and professional impact of ADHD symptoms in this population.

## Materials and methods

Study design

This was a cross-sectional, observational study based on an anonymous self-administered online questionnaire distributed to medical residents across multiple training centres in Portugal between March and June 2026. Participants were recruited using a non-probability, voluntary convenience sampling strategy through online questionnaire distribution. No information regarding the specific training institution was collected to preserve participant anonymity.

Participants

Eligible participants were medical residents of any specialty currently undertaking specialty training in Portugal. Participants were recruited using a non-probability, voluntary convenience sampling strategy through electronic distribution via institutional hospital e-mail channels. As dissemination occurred through open electronic networks and the number of individual residents reached was not recorded, the number invited and the response rate could not be reliably determined. Informed consent was obtained electronically prior to questionnaire completion. No formal a priori sample size calculation was performed, as recruitment was exploratory and based on voluntary participation during the predefined study period. All questionnaire items were mandatory; therefore, incomplete questionnaires could not be submitted, and no incomplete responses were excluded. Duplicate submissions were prevented by restricting the Google Forms questionnaire (Google LLC, Mountain View, CA, USA) to one response per Google account. All completed questionnaires received during the recruitment period were included in the analysis, resulting in a final sample of 225 participants.

Data collection

Data were collected through an anonymous electronic questionnaire distributed online. The questionnaire included the following domains: (1) sociodemographic characteristics (age, sex, year of residency, and medical specialty); (2) self-reported diagnosed or suspected ADHD and whether a formal clinical evaluation had been performed; (3) medication use, including medication type, periods of use, motivations for use, and prescriber category; (4) self-reported psychiatric comorbidities, including anxiety disorders, depressive disorders, bipolar disorder, obsessive-compulsive disorder, post-traumatic stress disorder, insomnia or other sleep disorders, autism spectrum disorder, eating disorders, and substance use disorders; (5) perceived functional and professional impact of ADHD symptoms. The questionnaire was developed by the research team and was not formally pilot-tested or subjected to expert validation. The complete Portuguese-language questionnaire and its English translation are provided in Appendices A and B, respectively.

ADHD symptom screening

ADHD symptoms were assessed using the six-item ASRS-v1.1 screener, originally developed by Kessler et al. [11], using the Portuguese adaptation described by Mattos et al. [12]. The Portuguese version of the ASRS-v1.1 has not been specifically validated in Portuguese medical residents. The six items assess core adult ADHD symptoms, with four items predominantly addressing inattention and executive difficulties and two addressing hyperactivity/restlessness. Each item is answered independently using a frequency scale. For items 1-3, responses of “sometimes”, “often”, or “very often” were scored as 1, while “never” or “rarely” were scored as 0. For items 4-6, responses of “often” or “very often” were scored as 1, while “never”, “rarely”, or “sometimes” were scored as 0. Item scores were summed to obtain a total score ranging from 0 to 6, with a score of 4 or more considered a positive screen. No separate subscale scores were calculated. The ASRS-v1.1 is a screening instrument and does not establish a diagnosis.

Statistical analysis

Descriptive statistics were used to characterise the study sample and main outcome variables. Categorical variables were presented as absolute frequencies and percentages. Associations between categorical variables were assessed using Pearson's chi-square (χ²) test. Statistical significance was set at p < 0.05. All analyses were performed using IBM SPSS Statistics (version 29.0; IBM Corporation, Armonk, NY, USA).

Ethical considerations

The study protocol was approved by the Ethics Committee for Health of the Unidade Local de Saúde de Lisboa Ocidental (approval no. 2025-95). All participants were fully informed of the study's objectives and procedures prior to enrolment. Anonymity and data confidentiality were ensured throughout all stages of the study. No costs were incurred by participating institutions or individuals.

## Results

Sample characteristics

A total of 225 medical residents completed the questionnaire. The sample was predominantly female (n = 182; 80.9%), with most participants aged between 25 and 29 years (n = 153; 68.0%). Most participants were single (n = 161; 71.6%). Residency year was broadly distributed, with the largest groups in the second year (n = 55; 24.4%) and first year (n = 52; 23.1%). The most frequently represented specialties were pediatrics (n = 56; 24.9%), psychiatry (n = 54; 24.0%), and family medicine (n = 34; 15.1%). Full sociodemographic characteristics are presented in Table 1.

**Table 1:** Sociodemographic characteristics of the study sample (n=225)

Characteristic	Number (n)	Percentage (%)
Sex		
Female	182	80.9
Male	43	19.1
Age group		
< 25 years	1	0.4
25–29 years	153	68.0
30–34 years	62	27.6
> 34 years	9	4.0
Marital status		
Single	161	71.6
Married	35	15.6
Domestic partnership	28	12.4
Other	1	0.4
Year of residency		
1^st^ year	52	23.1
2^nd^ year	55	24.4
3^rd^ year	43	19.1
4^th^ year	50	22.2
5^th^ year	23	10.2
6^th^ year	2	0.9
Specialty (most represented)		
Pediatrics	56	24.9
Psychiatry	54	24.0
Family Medicine	34	15.1
Child and Adolescent Psychiatry	16	7.1
Other specialties	65	28.9

ADHD diagnosis and symptom screening

Eighty-four participants (37.3%) reported having received an ADHD diagnosis or believing that they had ADHD. Of these, 56 (66.7%) reported that ADHD was first identified after starting residency training, whereas five participants (6.0%) reported identification before the age of 18.

The most frequently reported pathway to recognising possible ADHD was self-directed research (n = 38; 45.2%), followed by symptom recognition by colleagues (n = 19; 22.6%) and referral following a medical or psychological consultation (n = 12; 14.3%). Notably, 47 participants (56.0%) reported no formal evaluation before reporting diagnosed or suspected ADHD.

Applying the ASRS-v1.1 screener to the full sample, 111 participants (49.3%) screened positively. Among those reporting diagnosed or suspected ADHD, 70 (83.3%) returned a positive screen, compared with 41 (29.1%) among those who did not report ADHD. Self-report and screening data are summarised in Table 2.

**Table 2:** Self-reported diagnosed or suspected ADHD and Adult ADHD Self-Report Scale version 1.1 (ASRS-v1.1) screening results (n=225)

Variable	Number (n)	Percentage (%)
Self-reported diagnosed or suspected ADHD	84	37.3
Age when ADHD was first identified (n=84)		
< 12 years	3	3.6
12-18 years	2	2.4
> 18 years, before residency	23	27.4
> 18 years, after starting residency	56	66.7
How ADHD was first suspected (n=84)		
Self-directed research	38	45.2
Symptom recognition by colleagues	19	22.6
Referral after medical/psychological consultation	12	14.3
Suggestion by family or friends	9	10.7
Other	6	7.1
Formal evaluation before reporting ADHD (n=84)		
No formal evaluation	47	56.0
Questionnaires in consultation	19	22.6
Psychological evaluation	11	13.1
Other	7	8.3
ASRS-v1.1 positive screen - total sample (n=225)	111	49.3
Among those reporting diagnosed or suspected ADHD (n=84)	70	83.3
Among those not reporting ADHD (n=141)	41	29.1

Medication use

A total of 58 participants (25.8%) reported lifetime use of ADHD medication. Stimulants, specifically methylphenidate or lisdexamfetamine, were the predominant pharmacological class, used by 57 participants (98.3% of users). Most medication users reported use during residency training (n = 47; 81.0%).

Primary motivations for medication use included formal medical indication (n = 40; 69.0%), therapeutic trials (n = 14; 24.1%), and cognitive enhancement (n = 16; 27.6%). Nineteen participants (32.8%) reported obtaining medication from a colleague or friend (n = 9; 15.5%), through self-prescription (n = 8; 13.8%), or without a formal prescription (n = 2; 3.4%).

Among those who had never used ADHD medication (n = 167), 57 (34.1%) had previously considered its use, while 46 (27.5%) indicated a willingness to consider pharmacological intervention only under medical recommendation.

Among participants reporting diagnosed or suspected ADHD, 57.1% (n = 48) had used medication, compared with 7.1% (n = 10) of those who did not report ADHD (χ² = 68.92, df = 1, p < 0.001). The latter group included 10 residents who reported using ADHD medication despite not reporting ADHD.

Medication use was significantly associated with medical specialty (χ² = 13.82, df = 4, p = 0.008). Psychiatry residents exhibited the highest prevalence of medication use (n = 24; 44.4%), compared with paediatrics (n = 9; 16.1%), family medicine (n = 8; 23.5%), child and adolescent psychiatry (n = 4; 25.0%), and the remaining specialties (n = 13; 20.0%). Medication use data are presented in Table 3.

**Table 3:** ADHD medication use patterns (n=225) *Percentages may sum to more than 100% as multiple options could be selected.

Variable	Number (n)	Percentage (%)
Ever used ADHD medication (n=225)	58	25.8
Type of medication (n=58)		
Stimulant (methylphenidate or lisdexamfetamine)	57	98.3
Non-stimulant only	1	1.7
Period of medication use (n=58)*		
During residency	47	81.0
During undergraduate studies	14	24.1
Before university	2	3.4
Objective of use (n=58)*		
Medical indication	40	69.0
Therapeutic trial	14	24.1
Cognitive enhancement (study/work)	16	27.6
Prescriber (n=58)		
Attending physician	39	67.2
Colleague or friend	9	15.5
Self-prescribed	8	13.8
Not formally prescribed	2	3.4
Among non-users (n=167): considered using medication		
Yes	57	34.1
Only if recommended by a physician	46	27.5
No	64	38.3

Psychiatric comorbidities

In the overall study population, the most prevalent psychiatric comorbidity was anxiety disorder (n = 92; 40.9%), followed by depressive disorder (n = 59; 26.2%) and sleep disturbances (n = 26; 11.6%). Absence of psychiatric comorbidity was reported by 42.2% of overall participants (n = 95), but in the self-reported ADHD subgroup, only 27.4% (n = 23) indicated no additional psychiatric diagnosis.

Participants in the self-reported ADHD subgroup were significantly more likely to report at least one comorbid condition (n = 61; 72.6% vs n = 69; 48.9%, p < 0.001), with a higher prevalence of sleep disturbances (n = 15; 17.9% vs n = 11; 7.8%, p = 0.022). Although anxiety and depressive disorders were numerically more frequent among those with ADHD, these differences were not statistically significant (anxiety: n = 41; 48.8% vs n = 51; 36.2%, p = 0.062; depression: n = 26; 31.0% vs n = 33; 23.4%, p = 0.213).

Functional impact

The perceived professional impact of ADHD was characterised as negative by the vast majority of affected residents (n = 70; 83.3%), whereas a positive impact was reported by only two participants (2.4%). The most frequently identified areas of functional impairment were concentration on bureaucratic tasks (n = 69; 82.1%), organisational skills and scheduling (n = 54; 64.3%), and concentration on clinical responsibilities (n = 45; 53.6%).

When asked whether identifying ADHD had helped improve professional performance, 36 participants (42.9%) responded affirmatively, 24 (28.6%) responded "perhaps", and 24 (28.6%) reported no professional benefit. Comorbidity and functional impact data are presented in Table 4.

**Table 4:** Psychiatric comorbidities and perceived professional impact in the total sample (n = 225) and self-reported ADHD subgroup (n = 84) *Percentages may sum to more than 100% as multiple options could be selected.

Variable	Number (n)	Percentage (%)
Psychiatric comorbidities: total sample (n=225)*		
Anxiety disorder	92	40.9
Depressive disorder	59	26.2
Sleep disturbance	26	11.6
Eating disorder	23	10.2
Obsessive-compulsive disorder	9	4.0
Autism spectrum disorder	5	2.2
No psychiatric comorbidity	95	42.2
Psychiatric comorbidities: ADHD subgroup (n=84)*		
Anxiety disorder	41	48.8
Depressive disorder	26	31.0
Sleep disturbance	15	17.9
Eating disorder	12	14.3
No psychiatric comorbidity	23	27.4
Perceived professional impact: ADHD subgroup (n=84)		
Negative	70	83.3
Neutral	12	14.3
Positive	2	2.4
Work domains most affected: ADHD subgroup (n=84)*		
Concentration on bureaucratic tasks	69	82.1
Schedule organisation	54	64.3
Concentration on clinical tasks	45	53.6
Performance on-call/emergency shifts	33	39.3
Relationships with colleagues	25	29.8
Identifying ADHD helped improve professional performance (n=84)		
Yes	36	42.9
Perhaps	24	28.6
No	24	28.6

The results of inferential analyses according to self-reported ADHD status are summarised in Table 5. The distribution of ADHD medication use across medical specialties and the corresponding overall chi-squared test are presented in Table 6.

**Table 5:** Clinical characteristics according to self-reported diagnosed or suspected ADHD χ², Pearson’s chi-square test; df, degrees of freedom; OR, odds ratio; CI, confidence interval. ORs represent the odds of each outcome among participants with self-reported diagnosed or suspected ADHD compared with participants without self-reported ADHD. Statistical significance was set at p < 0.05.

Variable	Total (N = 225), n (%)	Self-reported diagnosed or suspected ADHD (n = 84), n (%)	No self-reported ADHD (n = 141), n (%)	OR (95% CI)	χ²	df	p-value
Medication use	58 (25.8%)	48 (57.1%)	10 (7.1%)	17.47 (8.05–37.90)	68.92	1	<0.001
Any psychiatric comorbidity	130 (57.8%)	61 (72.6%)	69 (48.9%)	2.77 (1.55–4.95)	12.10	1	<0.001
Sleep disturbance	26 (11.6%)	15 (17.9%)	11 (7.8%)	2.57 (1.12–5.90)	5.21	1	0.022
Anxiety disorder	92 (40.9%)	41 (48.8%)	51 (36.2%)	1.68 (0.97–2.91)	3.48	1	0.062
Depressive disorder	59 (26.2%)	26 (31.0%)	33 (23.4%)	1.47 (0.80–2.69)	1.55	1	0.213

**Table 6:** Association between medical specialty and ADHD medication use Pearson’s chi-square test showed a significant association between medical specialty and ADHD medication use (χ² = 13.82, df = 4, p = 0.008). Percentages for medication use are calculated within each medical specialty.

Medical specialty	Total, n (%)	Medication use: Yes, n (%)	Medication use: No, n (%)
Psychiatry	54 (24.0%)	24 (44.4%)	30 (55.6%)
Paediatrics	56 (24.9%)	9 (16.1%)	47 (83.9%)
Family Medicine	34 (15.1%)	8 (23.5%)	26 (76.5%)
Child and Adolescent Psychiatry	16 (7.1%)	4 (25.0%)	12 (75.0%)
Other specialties	65 (28.9%)	13 (20.0%)	52 (80.0%)
Total	225 (100.0%)	58 (25.8%)	167 (74.2%)

## Discussion

In the present study, 37.3% (n = 84) of Portuguese medical residents reported having ADHD. By contrast, global estimates of ADHD prevalence in the general adult population are approximately 2.5-3.1% [13]. This figure cannot be interpreted as the prevalence of clinically confirmed ADHD and likely reflects substantial self-selection in a voluntary online survey on a personally salient topic. It may also reflect enhanced health literacy and the possibility that residency demands make pre-existing attentional and executive difficulties more noticeable. Among participants reporting ADHD, 66.7% (n = 56) indicated that the condition was first identified after starting residency. In a large UK cohort of 2,877 doctors presenting with mental health difficulties, Perera and Al-Najjar (2025) found that 35% screened positive for ADHD using the ASRS-v1.1, a figure markedly higher than population norms [14].

Of particular concern, 56.0% (n = 47) of participants reporting ADHD had undergone no formal evaluation, with self-directed research (n = 38; 45.2%) and symptom recognition by colleagues (n = 19; 22.6%) identified as the main pathways to recognition. The high rates of comorbid anxiety (n = 41; 48.8%) and depression (n = 26; 31.0%), both of which may contribute to attentional complaints, further highlight the risk of self-attribution in the absence of structured clinical assessment.

The significant association between self-reported ADHD and any psychiatric comorbidity (n = 61; 72.6% vs n = 69; 48.9%; p < 0.001) is consistent with findings from Perera and Al-Najjar (2025), who reported that, among doctors screening positive for ADHD, 46% also scored above threshold for depression and 44% for anxiety and cautioned against diagnostic overshadowing in this population [14].

Medication use was reported by 25.8% (n = 58) of participants, with stimulants accounting for nearly all reported pharmacological interventions. Of the medication users, 32.8% (n = 19) obtained medication from a colleague or friend, through self-prescription, or without a formal prescription, a pattern broadly consistent with reports from Israel and the United States [15-17]. Medication use was associated with specialty (p = 0.008), and psychiatry residents reported the highest rate (n = 24; 44.4%). Although professional familiarity with psychiatric pharmacology might contribute to this finding, the cross-sectional data do not establish the underlying reason. Ten participants used ADHD medication despite not reporting ADHD; however, the motivations of these specific participants were not explored. These prescribing pathways warrant attention because they may occur without independent assessment or longitudinal monitoring.

The perceived professional impact of ADHD was characterised as negative by 83.3% (n = 70) of participants in the self-reported ADHD group, with concentration on bureaucratic tasks (n = 69; 82.1%), organisational skills (n = 54; 64.3%), and concentration on clinical responsibilities (n = 45; 53.6%) being the most frequently reported areas of difficulty. These findings are consistent with reports that residents and fellows with ADHD referred for performance difficulties commonly experience challenges involving organisational efficiency, engagement with training requirements, and professional performance [7].

These findings may have implications for postgraduate medical training programmes. Residents experiencing attentional, organisational, or performance difficulties may benefit from access to structured and confidential clinical assessment, rather than responses focused solely on performance or disciplinary concerns. Training institutions may also consider providing clearer pathways for referral, guidance regarding appropriate prescribing practices, and individualised support when functional difficulties are identified. Given the frequency of psychiatric comorbidity observed in this study, assessment should also consider alternative or co-occurring conditions that may contribute to attentional symptoms. Targeted remediation programmes incorporating a global biopsychosocial assessment and tailored interventions have been described for residents and fellows with performance concerns [18]. The findings also raise concerns regarding medication access outside conventional prescribing pathways, although interpretation is limited, as the questionnaire did not assess the specific circumstances of non-prescribed medication use.

Strengths and limitations

This study has several strengths. The use of a standardised ADHD screening instrument alongside an anonymous questionnaire allowed us to examine self-identification of possible ADHD symptoms during medical residency, while simultaneously addressing medication use, psychiatric comorbidity, and perceived professional impact. The inclusion of residents from multiple specialties and the assessment of medication access outside conventional prescribing pathways add practical relevance to the findings.

The study also has several limitations. Its cross-sectional design and reliance on self-report data preclude causal inference, while reliance on self-report data may introduce reporting and recall bias. The questionnaire combined participants who reported having received a diagnosis with those who believed that they had ADHD; consequently, the proportion reporting diagnosed or suspected ADHD (n = 84; 37.3%) cannot be interpreted as the prevalence of clinically confirmed ADHD. The ASRS-v1.1 is a screening instrument rather than a diagnostic tool. The voluntary online recruitment method creates a substantial risk of self-selection bias, and the effective response rate could not be determined reliably. Participation bias, health literacy, and recruitment through resident networks may also have influenced the observed proportion. The absence of an a priori sample-size calculation and a defined sampling frame further limits the precision and generalisability of the findings. Associations were based on unadjusted analyses, and residual confounding by demographic, training-related, or psychiatric variables cannot be excluded. No correction for multiple comparisons was applied; therefore, inferential findings should be interpreted cautiously. Finally, the overrepresentation of psychiatry and paediatrics residents limits generalisability to the broader medical residency population.

## Conclusions

Portuguese medical residency programmes may benefit from accessible, non-stigmatising diagnostic pathways and institutional support. Residents who suspect ADHD should have access to independent formal evaluation and clear guidance regarding the risks of self-prescription and medication use without appropriate assessment and follow-up. The high burden of psychiatric comorbidity in the self-reported ADHD group suggests that these residents may have complex clinical profiles requiring specialist evaluation rather than self-management.

Future research should employ structured diagnostic assessments, longitudinal designs, probability-based or broader recruitment strategies, and more balanced specialty representation to characterise ADHD prevalence, impact, and treatment outcomes in this population more accurately.

## Appendices

Appendix A

Original Survey

The following survey was administered in Portuguese to Portuguese-speaking medical residents.

Consentimento informado

Bem-vindo(a) ao estudo sobre a Perturbação de Hiperatividade e Défice de Atenção (PHDA) entre internos de formação específica. Este questionário, com duração aproximada de 5 minutos, tem como objetivos avaliar a prevalência do diagnóstico de PHDA autorreportado em internos de formação específica em Portugal, bem como caracterizar o padrão de utilização de medicação, a presença de comorbilidades psiquiátricas associadas e o impacto percecionado no desempenho profissional.

A tua participação é voluntária. Podes desistir a qualquer momento, sem qualquer prejuízo pessoal ou profissional. As tuas respostas serão tratadas com total confidencialidade, de forma anónima, e utilizadas exclusivamente para fins de investigação científica.

Se tiveres dúvidas ou precisares de mais informações, podes contactar os Investigadores Principais através dos seguintes contactos: E-mail: estudo.phda.ifes@gmail.com; Tel. (Secretariado do Serviço de Psiquiatria da ULSLO): 210 432 721.

Para questões relacionadas com a proteção dos teus dados pessoais, podes contactar o Encarregado de Proteção de Dados da ULSLO através do e-mail dpo@ulslo.min-saude.pt.

Ao clicares em "Seguinte" para continuar o questionário, estás a confirmar que compreendeste a informação acima e que aceitas participar livremente neste estudo.

Os Investigadores Principais: João Pedro Azenha, Médico Interno de Psiquiatria, cédula n.º 69936; Margarida Cândido, Médica Interna de Psiquiatria, cédula n.º 74264.

Dados sociodemográficos

Sexo: Masculino / Feminino / Outro

Idade: < 25 anos / 25-29 anos / 30-34 anos / > 34 anos

Estado civil: Solteiro(a) / Casado(a) / União de Facto / Outro

Especialidade: (lista completa de especialidades médicas portuguesas)

Ano de internato: 1.º ano / 2.º ano / 3.º ano / 4.º ano / 5.º ano / 6.º ano

Uso de medicação para PHDA

Alguma vez utilizaste medicação para a PHDA? Sim / Não

Se nunca utilizaste medicação para a PHDA, já consideraste fazê-lo? Sim / Não / Apenas se fosse recomendado por um médico

Que tipo de medicação? (seleciona todas as aplicáveis): Psicoestimulante (metilfenidato, lisdexanfetamina) / Não estimulante (atomoxetina, clonidina, guanfacina, bupropiom)

Em que momento? (seleciona todas as aplicáveis): Antes da faculdade / Durante a faculdade / Durante o internato

Com que frequência utilizas ou utilizaste a medicação? (seleciona todas as aplicáveis): Ocasionalmente ("on demand") / Em períodos de preparação para exames / Regularmente (uso diário)

Com que objetivo? (seleciona todas as aplicáveis): Por indicação médica / Como prova terapêutica / Uso recreativo / Para potenciar o estudo / Para potenciar o trabalho

Quem prescreveu a medicação? Médico assistente / Colega ou amigo / Eu próprio(a) / Não foi prescrita

A medicação foi eficaz? (escala de 1 a 5, em que 1 = Não ajudou e 5 = Muito eficaz)

História de diagnóstico de PHDA

Alguma vez recebeste ou pensas ter o diagnóstico de PHDA? Sim / Não

Em que idade foste diagnosticado(a) pela primeira vez? < 12 anos / 12-18 anos / > 18 anos e antes de iniciar o internato / > 18 anos e depois de iniciar o internato

Como percebeste que poderias ter PHDA? Pesquisei sobre o tema por conta própria / Amigos ou familiares sugeriram / Sintomas relatados por colegas de trabalho / Recomendaram-me avaliação após consulta médica/psicológica / Outra

Fizeste alguma avaliação formal antes do diagnóstico? Não fiz nenhuma avaliação formal / Avaliação psicológica / Questionários preenchidos em consulta / Outra

Impacto na vida profissional

Como descreverias o impacto da PHDA na tua vida profissional como interno? Positivo / Negativo / Neutro

Que aspetos da tua rotina são mais afetados pelos sintomas da PHDA? (seleciona todas as aplicáveis): Organização de horários / Concentração em tarefas clínicas / Concentração em tarefas burocráticas / Desempenho em turnos ou serviço de urgência / Relacionamento com colegas

Achas que o diagnóstico ajudou a melhorar o teu desempenho profissional? Sim / Não / Talvez

Comorbilidades psiquiátricas

Tens ou já tiveste outros diagnósticos psiquiátricos? (seleciona todos os aplicáveis): Ansiedade / Depressão / Perturbação Afetiva Bipolar / Perturbação Obsessivo-Compulsiva / Perturbação Pós-Stress Traumático / Insónia ou outras perturbações do sono / Perturbações do Espetro do Autismo / Perturbação do Comportamento Alimentar / Perturbação do Uso de Substâncias / Nenhuma

Escala de Autoavaliação da perturbação de hiperatividade e défice de atenção para o adulto v1.1 (ASRs-v1.1)

O questionário abaixo pode ser usado como um ponto de partida para te ajudar a reconhecer os sinais/sintomas da PHDA na idade adulta. Se responderes "frequentemente" ou "muito frequentemente" a 4 ou mais das 6 questões, isso poderá indicar um resultado positivo no rastreio. Contudo, não configura um diagnóstico nem substitui uma consulta com um profissional com conhecimento e experiência na área.

Opções de resposta para todas as questões: Nunca / Raramente / Algumas vezes / Frequentemente / Muito frequentemente

Nos últimos 6 meses com que frequência sentiste dificuldade em finalizar os detalhes de um projeto, após terminar as partes mais desafiantes?

Nos últimos 6 meses com que frequência sentiste dificuldade em pôr as coisas em ordem quando tens de executar uma tarefa que exige organização?

Nos últimos 6 meses com que frequência sentiste dificuldade em lembrares-te de compromissos ou obrigações?

Nos últimos 6 meses com que frequência evitaste ou adiaste uma tarefa que exija muita concentração?

Nos últimos 6 meses com que frequência ficaste inquieto(a) ou mexeste repetidamente as mãos e os pés, quando tens de estar sentado(a) durante um longo período de tempo?

Nos últimos 6 meses com que frequência te sentiste excessivamente ativo(a) e compelido(a) a fazer coisas, como se estivesses "ligado(a) à corrente"?

Appendix B

English Translation of the Survey

The following is an English translation of the original Portuguese survey. This English version was prepared for reporting purposes only and was not administered to participants.

Informed consent

Welcome to the study on Attention-Deficit/Hyperactivity Disorder (ADHD) among medical residents undergoing specialty training. This questionnaire, which takes approximately five minutes to complete, aims to assess the prevalence of self-reported ADHD diagnosis among medical residents undergoing specialty training in Portugal, as well as to characterise patterns of medication use, the presence of associated psychiatric comorbidities, and the perceived impact on professional performance.

Your participation is voluntary. You may withdraw at any time without any personal or professional disadvantage. Your responses will be treated with complete confidentiality and anonymity and will be used exclusively for scientific research purposes.

If you have any questions or require additional information, you may contact the Principal Investigators using the following contact details: E-mail: estudo.phda.ifes@gmail.com; Tel. (Secretariat of the Psychiatry Department, ULSLO): 210 432 721.

For matters related to the protection of your personal data, you may contact the Data Protection Officer of ULSLO at dpo@ulslo.min-saude.pt.

By clicking “Next” to continue with the questionnaire, you confirm that you have understood the information above and freely agree to participate.

Principal Investigators: João Pedro Azenha, Psychiatry Resident, Medical License No. 69936 Margarida Cândido, Psychiatry Resident, Medical License No. 74264

Sociodemographic data

Sex: Male / Female / Other

Age: < 25 years / 25-29 years / 30-34 years / > 34 years

Marital status: Single / Married / Domestic partnership / Other

Specialty: (complete list of Portuguese medical specialties)

Year of residency: 1st year / 2nd year / 3rd year / 4th year / 5th year / 6th year

ADHD medication use

Have you ever used medication for ADHD? Yes / No

If you have never used ADHD medication, have you ever considered using it? Yes / No / Only if recommended by a physician

What type of medication? (select all that apply): Stimulant (methylphenidate, lisdexamfetamine) / Non-stimulant (atomoxetine, clonidine, guanfacine, bupropion)

At what stage did you use it? (select all that apply): Before university / During university / During residency

How frequently do you use or have you used the medication? (select all that apply): Occasionally (“on demand”) / During periods of examination preparation / Regularly (daily use)

For what purpose? (select all that apply): Medical indication / As a therapeutic trial / Recreational use / To enhance studying / To enhance work performance

Who prescribed the medication? Attending physician / Colleague or friend / Self-prescribed / Not prescribed

Was the medication effective? Scale from 1 to 5, where 1 = Did not help and 5 = Very effective

History of ADHD diagnosis

Have you ever received an ADHD diagnosis or do you believe that you have ADHD? Yes / No

At what age were you first diagnosed? < 12 years / 12-18 years / > 18 years and before starting residency / > 18 years and after starting residency

How did you first realise that you might have ADHD? I researched the topic on my own / Friends or family suggested it / Symptoms were pointed out by work colleagues / I was advised to undergo an assessment following a medical/psychological consultation / Other

Did you undergo any formal assessment before the diagnosis? I did not undergo any formal assessment / Psychological assessment / Questionnaires completed during a consultation / Other

Impact on professional life

How would you describe the impact of ADHD on your professional life as a medical resident? Positive / Negative / Neutral

Which aspects of your routine are most affected by ADHD symptoms? (select all that apply): Schedule organisation / Concentration on clinical tasks / Concentration on bureaucratic tasks / Performance during on-call or emergency shifts / Relationships with colleagues

Do you think that receiving the diagnosis helped improve your professional performance? Yes / No / Perhaps

Psychiatric comorbidities

Do you currently have, or have you ever had, any other psychiatric diagnoses? (select all that apply): Anxiety / Depression / Bipolar Disorder / Obsessive-Compulsive Disorder / Post-Traumatic Stress Disorder / Insomnia or other sleep disorders / Autism Spectrum Disorder / Eating Disorder / Substance Use Disorder / None

Adult ADHD Self-Report Scale v1.1 (ASRS-v1.1)

The questionnaire below may be used as a starting point to help you recognise signs/symptoms of ADHD in adulthood. If you answer “often” or “very often” to four or more of the six questions, this may indicate a positive screening result. However, it does not constitute a diagnosis and does not replace consultation with a professional with knowledge and experience in this field.

Response options for all questions: Never / Rarely / Sometimes / Often / Very often

In the past six months, how often have you had difficulty finishing the final details of a project, once the challenging parts have been completed?

In the past six months, how often have you had difficulty getting things in order when you have to perform a task that requires organisation?

In the past six months, how often have you had difficulty remembering appointments or obligations?

In the past six months, how often have you avoided or delayed starting a task that requires a lot of concentration?

In the past six months, how often have you felt restless or repeatedly moved your hands and feet when you had to remain seated for a long period of time?

In the past six months, how often have you felt overly active and compelled to do things, as if you were “driven by a motor”?
